# Artisanal Extraction and Traditional Knowledge Associated with Medicinal Use of Crabwood Oil (*Carapa guianensis* Aublet.) in a Peri-Urban Várzea Environment in the Amazon Estuary

**DOI:** 10.1155/2016/5828021

**Published:** 2016-07-10

**Authors:** Mariane Nardi, Ana Cláudia Lira-Guedes, Helenilza Ferreira Albuquerque Cunha, Marcelino Carneiro Guedes, Karen Mustin, Suellen Cristina Pantoja Gomes

**Affiliations:** ^1^Programa de Pós Graduação em Biodiversidade Tropical, Universidade Federal do Amapá, Rodovia Juscelino Kubitschek, Km 2, Jardim Marco Zero, 68903-419 Macapá, AP, Brazil; ^2^Embrapa Amapá, Rodovia Juscelino Kubitschek, 2600, Universidade, 68903-419 Macapá, AP, Brazil; ^3^Universidade do Estado do Amapa, Avenida Procópio Rola 1346, Centro, 68900-081 Macapá, AP, Brazil

## Abstract

Várzea forests of the Amazon estuary contain species of importance to riverine communities. For example, the oil extracted from the seeds of crabwood trees is traditionally used to combat various illnesses and as such artisanal extraction processes have been maintained. The objectives of this study were to (1) describe the process involved in artisanal extraction of crabwood oil in the Fazendinha Protected Area, in the state of Amapá; (2) characterise the processes of knowledge transfer associated with the extraction and use of crabwood oil within a peri-urban riverine community; and (3) discern medicinal uses of the oil. The data were obtained using semistructured interviews with 13 community members involved in crabwood oil extraction and via direct observation. The process of oil extraction is divided into four stages: seed collection; cooking and resting of the seeds; shelling of the seeds and dough preparation; and oil collection. Oil extraction is carried out within the home for personal use, with surplus marketed within the community. More than 90% of the members of the community involved in extraction of crabwood oil highlighted the use of the oil to combat inflammation of the throat. Knowledge transfer occurs via oral transmission and through direct observation.

## 1. Introduction

Lowland floodplain forests (hereafter referred to as várzea) of the Amazon estuary have their own particular dynamic, characterised by twice daily tidal inundations seen up to Óbidos, 870 km inland [1]. These inundations carry sediments into the forest, enriching the soil [2], and as the tide falls, the water takes seeds and other biological material from the forest and disperses them throughout the estuary. Many of these fruits and seeds are then collected by local people and used for various purposes, including medicinal uses. Beyond such local exploitation, many species of Amazonian biodiversity have drawn international interest. Increasing numbers of companies and industries based all over the world are turning their eyes to the Amazon in search of new resources, particularly essential oils which have cosmetic and physiotherapeutic potential.

One várzea species important to local riverine communities is the crabwood tree,* Carapa guianensis* Aublet. Crabwood is a mid- to large-sized tree, reaching 25 to 35 metres [3]. The species occurs at higher densities in várzea but is also found in upland forests (hereafter referred to as terra firme) of the Amazon [3]. The fruits are spherical and contain between 4 and 16 seeds which are released when the fruit falls and hits the ground [3, 4]. The wood is often used in the construction of houses and boats within riverine communities [5] and the seeds are used to extract oil, which has been used variously as a medicine, an ingredient in soap-making and to illuminate houses [6, 7]. In some Amazonian communities it has been reported that the process of artisanal oil extraction involves all family members, from children to the elderly and both men and women but that the bulk of the process is carried out by women [6, 8]. Knowledge of the extraction processes is reported to be passed orally and through direct observation [6, 8], though it has been noted that the involvement of young people may be declining, potentially endangering the survival of the practice [8]. While the details of the extraction process inevitably vary between communities, the overall process has generally been observed to consist of seed collection and storage; cooking and shelling of the seeds to prepare a dough; and oil extraction from the shaped dough [6–8]. Problems identified for the commercialisation of this artisanal crabwood oil have included community members lack of specific qualifications, a lack of organisation among the community members involved in extraction, and difficulties getting the product into the market [6], as well as the relatively time-consuming and labour-intensive methods involved in the extraction process [7].

Additionally, UNESCO have highlighted the need to consider culture within the process of product development, recognising traditional knowledge when it is being used [9]. The scientific literature has also increasingly recognised and validated traditional knowledge, principally through participative research aiming to build effective conservation actions [10–14]. Bringing to the forefront traditional groups and the knowledge they hold helps to both legitimise and protect such knowledge. However, the publication of traditional knowledge should be done in a responsible way, with consent of the community and giving credit where it is due. Founded in this, the objectives of this study were to (1) describe the process involved in artisanal extraction of crabwood oil, carried out by community members in the Fazendinha Protected Area (hereafter referred to as Fazendinha PA), in the state of Amapá; (2) characterise the processes of knowledge transfer associated with the extraction and use of crabwood oil within a peri-urban riverine community; and (3) discern medicinal uses of the oil.

## 2. Methods

### 2.1. Study Area

**Figure 1:**
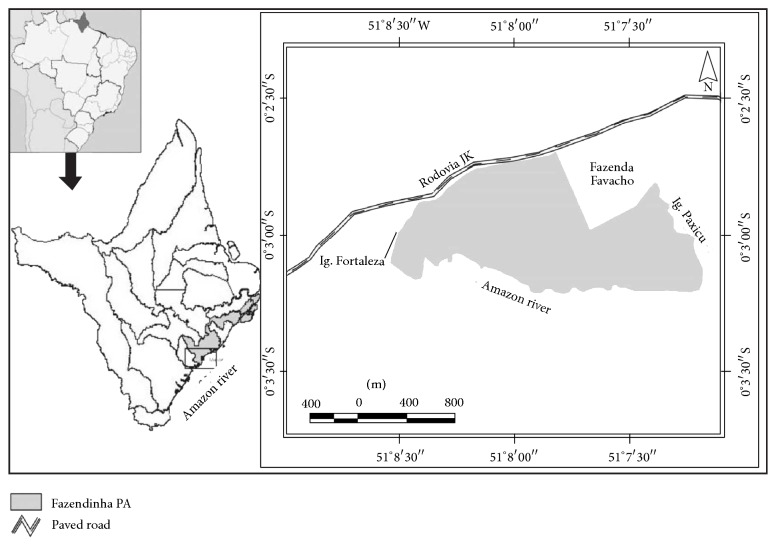
Map of the study area (Fazendinha PA) with insets showing its location within the state of Amapá and the location of the state within Brazil [15].

The study was carried out in the Fazendinha PA (Figure 1), located at 00°03′10,39′′S and 051°07′41,78′′W, approximately 15 km from the urban centre of Macapá, the state capital with a reported population in 2010 of 398 204 [16], and approximately 8 km from the centre of Santana, the 2nd largest conurbation in the state, with a reported population in 2010 of 101 262 [16]. The Fazendinha PA was created on 31st December 2004 [17] and has an area of 136.52 ha.

### 2.2. Methodological Procedures

As part of a related study, socioeconomic questionnaires were applied in 84 residences, representing approximately 30% of the total number of households in the study area [15]. Through these questionnaires and subsequently through snowball sampling based on suggestions made by some of the respondents, twenty-four community members were identified as being involved in crabwood oil extraction, though this is a subsample of the total number of people involved in such activities within the community. Of the 24 community members identified, thirteen were eventually interviewed and/or observed during the crabwood oil extraction process, as the other 11 community members could not be contacted or declined to participate in the study.

Data collection took place in 2012 using (1) semistructured interviews [18], based on questionnaires adapted from [8], and (2) through direct observation of the crabwood oil extraction process including a photographic register. All 13 interviewees signed the Terms of Consent, and all relevant permissions to collect traditional knowledge were obtained (see Acknowledgements).

The data collected on the process of crabwood oil production, knowledge transfer, and traditional uses of the oil are largely qualitative and as such no formal analyses were carried out. However, to support some statements regarding the prevalence of certain practices and beliefs, or the demographics of participants, percentages of respondents and sample means were calculated.

## 3. Results and Discussion

### 3.1. Socioeconomic Characteristics of Community Members Involved in Crabwood Oil Extraction

The community members involved in crabwood oil extraction in the Fazendinha PA ranged in age from 25 to 73 years old, with an average age of 54, and nine of the 13 aged 50 or more. They had lived in the community between 10 and 40 years. In line with previous studies in the Amazon estuary [6, 8], we found that crabwood oil extraction is predominantly conducted by women (12 of the 13 interviewees (92%) were female). The main income source for 34% of the 13 interviewees was a pension, and welfare benefits were also an important contribution to family income for 15% of interviewees. Beyond this, all inhabitants practice at least one subsistence activity, with shrimp fishing and açai collection being the most common. Community members involved in crabwood oil extraction differ from the other inhabitants of the protected area in terms of their lifestyle in relation to the environment and in general show many more traditional characteristics. The on-going habit of crabwood oil extraction is, in itself, indicative of preservation of an identity which is intimately linked to the environment.

In the Fazendinha PA, oil extraction is an activity in which family members work together, but in contrast to some other Amazonian communities, such as in the Tapajós National Forest in the state of Pará [19], there is no kind of community association for this type of work. Indeed, in the state of Amapá one of the difficulties facing traditional groups, such as those involved in extractive activities, riverine communities, artisanal fisherman, and others, is a lack of organisation into units which they themselves are recognised to be “traditional groups.” This is problematic as one of the prerequisites to be recognised as a traditional group by public policy mechanisms, in accordance with decree 6.040/2007 that instituted the National Policy for the Sustainable Development of Traditional Peoples and Communities [20], is self-recognition as such.

### 3.2. The Process of Artisanal Crabwood Oil Extraction

In the study community the full process of artisanal crabwood oil production can take between 30 and 90 days depending on the method of each individual and can be divided into the following four stages: (1) collection of the seeds; (2) cooking and resting of seeds; (3) shelling and dough preparation; and finally (4) oil collection. Resting of the seeds and oil collection are the most time-consuming stages. In the following four sections we describe each of these stages in turn and discuss our findings in relation to other studies of artisanal crabwood oil production in Amazonian communities.

#### 3.2.1. Seed Collection

**Figure 2:**
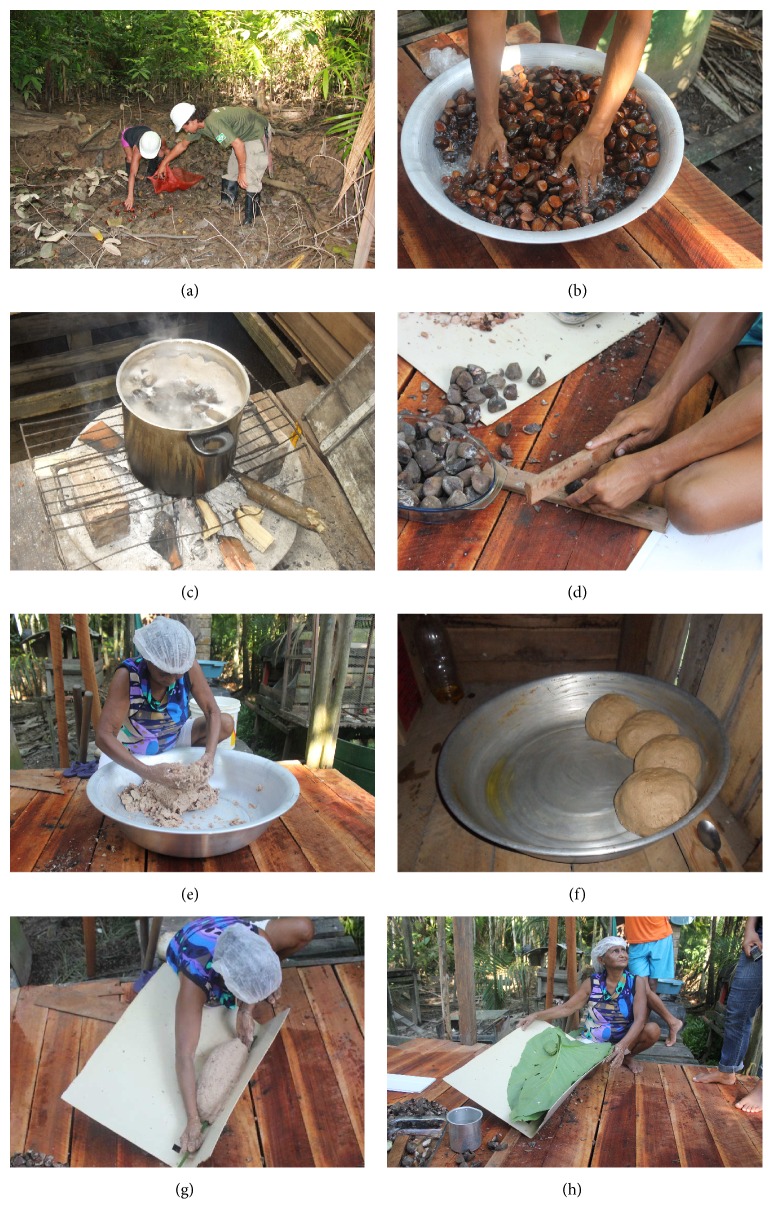
Stages of artisanal crabwood oil extraction in the Fazendinha PA, Macapá, Amapá, Brazil: (a) seed collection from the ground, underneath a crabwood tree; (b) washing seeds with clean water; (c) cooking seeds in an aluminium pan on a wood-burning stove; (d) shelling the seeds after 30-day resting; (e) kneading the dough; (f) oil collection from dough formed into balls; (g) dough in the form of a “loaf” with one of the community members creating a hole in the dough using a splint from an açai palm; (h) dough covered with a leaf from an aninga plant. Photos taken by Mariane Nardi Santos in 2012.

The interviewees identified the seed collection period as between March and July, and some reported that the seeds which fall in June are of higher quality and produce more oil. This coincides with the main period of seed production in the region, between March and June, with peak production in May [21, 22]. Two interviewees reported that there are different types of crabwood present in the Fazendinha PA. One stated that the difference is in the colour of the seeds, whereas the other stated that the difference is in seed size. However, the records in the area are all of* Carapa guianensis* Aublet. [23]. It was reported that seeds are collected from the ground during low tide, either from under the trees, or where seeds have accumulated after being carried by the falling tide (Figure 2(a)) and directly from the water during high tide, where seeds have accumulated with pieces of wood, tree trunks, leaves, or other seeds which are carried by the current. This stage of the oil production process requires a sound knowledge of the local environment. The same two collection practices have also been observed on the island of Juba in the state of Pará [6].

While it has been reported that in plantation areas a single person can collect between 200 and 300 kg of crabwood seeds per day [7], in reality the activity is not intensive as the seeds are collected only for community members own use. When encountered outside of organized collections seeds are also collected, particularly when the rising and falling tide waters carry them out of the forest and deposit them close to the homes of community members. In this case, the other stages of the extraction process only begin when sufficient seeds have been accumulated, with the interviewees citing half a container full as being sufficient to proceed with oil extraction. In these cases, only women and children are responsible for seed collection, in common with communities on the island of Juba [6]. Other interviewees reported that seed collection is an intensive activity which happens just once a year, and in these cases it is usually the men who participate in seed collection, owing to the heavy physical nature of the work, with age and health also cited as playing a role in which family members participate. This is similar to the reported collection activities in the Tapajós National Forest, also in the state of Pará, where approximately 30 women collect and transport between 1000 and 1500 kg of seeds in a single day, with men helping during transport [19]. This type of en-masse collection usually occurs once a year, sometimes only every other year, depending on seed availability and the quantity of oil already stored in the household. Interestingly, four of the 13 interviewees reported that they do not collect seeds in the Fazendinha PA but rather bring the seeds in from other places, and two of them actually buy the seeds to produce the crabwood oil. This result shows the importance of continuing the practice of oil extraction for these community members, even when the raw material cannot be obtained directly from their environment. That being said, the crabwood seeds are in fact an abundant resource in the study area [24], and the practice of accessing seeds from other areas results from a lack of knowledge in relation to their resource use rights and a fear of being fined by the authorities which enforce the rules of use of the protected area. Better communication between managing authorities and local people could help to combat this type of misunderstanding and improve relationships between the two groups, ultimately leading to more sustainable conservation of the protected area.

Raffia sacks (Figure 2(a)) and baskets are usually used for seed collection, and the seeds are also stored, for variable amounts of time, in open containers to avoid the seeds germinating due to humidity. When the seeds are germinating they can sometimes no longer be used, with the decision made based on the size of the sprout (the smaller the sprout the more likely the seed will still be used). Seed quality can also be compromised by larvae of* Hypsipyla* sp., with* Hypsipyla ferrealis* being the most abundant species in the state of Amapá [25]. Seeds considered to be rotten or very soft or which have been gnawed by animals are discarded throughout the process, including the time during seed collection. Depending on the report, around 40–50% of seeds may need to be discarded, mainly as a result of infestation by* Hypsipyla* larvae [19, 25, 26]. In Tomé-Açú in the state of Pará, after collection the seeds are placed in water tanks and spoiled seeds are removed [7]. In the state of Amazonas, the oil extractors conduct a preselection during the collection process, discarding seeds gnawed by animals, infested with* Hypsipyla*, desiccated or with a very dark shell, and germinated seeds are only considered suitable for processing for oil extraction if the sprout is 2 cm or less [8]. To avoid losses of yield and reductions in oil quality, after collection ideally the seeds would be left in water for 24 hours to eliminate larvae of* Hypsipyla* sp. and would then be cooked shortly afterwards [3, 26, 27]. However, such precautions were not seen to be taken by the community members of the Fazendinha PA.

#### 3.2.2. Cooking and Resting of Seeds

Interviewees reported that before being cooked the seeds are washed in clean water (Figure 2(b)), usually river water of which inhabitants treat themselves with hypochlorite, as water supply to the area is poor. The seeds are then cooked in available containers (butter cans, paint tins, or aluminium pans), on a wood-burning stove (Figure 2(c)), as wood is readily available from the forest and therefore costs and time associated with cooking are minimised. These findings are in common with those reported from parts of the states of Amazonas [8] and Pará [6]. This is another stage of the process in which men are involved [6] as this is also a task that requires strength, mainly due to the use of inadequate cooking vessels. Reported seed cooking times varied between interviewees, from 15 minutes to 7 hours, and were most commonly reported to be between 30 minutes and 2 hours. These findings are consistent with a study carried out in the state of Amazonas by Mendonça and Ferraz [8] where the cooking time was reported to be between one and three hours. However, in direct observations made in the Fazendinha PA, the cooking time was approximately 40 minutes after reaching boiling point. Both in the study area, and in Amazonas [8], it is common to remove a seed during cooking and try to break the shell to check the texture of the seed and therefore the stage of cooking.

Interviewees stressed the importance of resting of the cooked seeds, which fits with findings that the cell wall is modified during this resting period, allowing the oil to be extracted more easily from the seed [8]. The seeds are left in ventilated containers, most commonly baskets or raffia sacks, in a place with protection from rain and direct sunlight, for example, in a dark place within the house. The containers in which the seeds are stored are covered with leaves of aninga or açai, according to 53.8% of the informants. Resting usually lasts for a period of 30 days, often marked in a calendar, though it can be as little as 15 days, according to interviewees. These reported resting times are similar to those in other regions between 10 to 15 [7] and 15 to 40 days [28]. Some interviewees test the seeds by opening one and pressing the flesh; if it yields oil the seeds are ready to pass to the next stage of the extraction process.

Seeds can begin to ferment during the resting stage, and the appearance of fungus on the shells as a result of humid storage conditions has been reported [8]. Nine of the interviewees reported the presence of fungus during this stage of the process, though they reported that these seeds are only discarded when they are in an advanced state of degradation. These signs of fermentation can give their own different characteristics to oil extracted artisanally, and some interviewees believe that artisanally extracted oil has more medicinal properties than industrially produced (pressed) oil. Indeed, researchers have found active substances in oil which was subjected to this fermentation stage that do not appear in oil extracted by other methods [29].

#### 3.2.3. Shelling of Seeds and Dough Preparation

Following information provided by the interviewees, after being rested the seeds are broken (Figure 2(d)), usually with a piece of wood, though small hammers and knives are also used. The flesh is then removed using a small aluminium, or home-made wooden spoon. Much care is taken to avoid waste during removal of the shell. As with seed collection, this stage of the process involves a larger number of family members collaborating to carry out a relatively laborious part of the process of oil extraction. Interviewees reported that during this stage more seeds are excluded as a result of being hard or rotten, or infested with fungus. However, during direct observations of the process few seeds were removed during this stage, and even larvae of* Hypsipyla* sp. remained in the dough. Indeed, while one interviewee reported that a lack of selectivity of the seeds can compromise oil production, five admitted that they do not perform any such selection at this stage of the extraction process.

After shelling, the flesh is softened by hand and kneaded into a homogenous dough (Figure 2(e)). This differs from the practices of extractors on the island of Juba in the state of Pará, who press the seeds using their feet [6]. There is some evidence that these practices can change over time, with one community in the state of Amazonas pressing the seeds using their feet in 1996, while in 2004 in the same community the dough was seen to be kneaded by hand [8]. This kneading stage and subsequent oil collection (see Section 3.2.4) are strictly restricted to one or two people of a certain skill level, and substitutions are only allowed when there is some restriction on participation due to social rules associated with crabwood oil extraction. While these rules may be generalised within the community or restricted to a few or even just one family, there were two general rules which were agreed upon by all interviewees: (1) menstruating women should not touch or even look at the dough, as this can stop the flow of oil; (2) people with “olho ruim” (bad look) or “bad” people should not see these stages of the process. These rules are widespread in the Amazon and form part of the social rules established over time to control and explain possible problems that can occur in the oil extraction process. However, the exact form of these rules varies between communities and regions. On the island of Juba it is believed that menstruating and postpartum women should not handle the dough, nor should a person in mourning [6]. One interviewee in the Fazendinha PA similarly stated that following the death of a relative one should not touch the dough. In Amazonas it is believed that pregnant and menstruating women should be prevented from looking at or touching the dough [8], and five interviewees in the Fazendinha PA also stated a belief that pregnant women should not participate in this part of the process. In both Amazonas and on Juba community members state the need to screen the dough from the sight of “jealous people” [6, 8]. There is a risk that policies for crabwood oil extraction will not be accepted by local communities if these social rules are not incorporated.

#### 3.2.4. Oil Collection

The interviewees reported two different methods of shaping the dough: into a ball or into a “loaf.” When the dough is formed into a ball (Figure 2(f)) it is placed in the higher part of an inclined container, and the oil runs down into the lower part. The oil is then collected every day using a spoon and is stored in a sealed bottle to prevent any residues entering the oil. When the dough is formed into a loaf (Figure 2(g)) it is placed in a “biqueira,” which is an improvised tool with two straight sides forming a 90° angle, positioned in an inclined manner so that the oil can run down the groove and fall into a container positioned below. To make the process more efficient, a splint (usually made from a palm leaf) is used to make a hole in the dough in line with the junction of the two parts of the “biqueira” to facilitate the release of the oil. Some extractors also cover the dough with aninga leaves (Figure 2(h)) and/or use a cotton wick to direct the flow of oil from the loaf of dough. Previously wooden “biqueiras” were frequently used in more isolated communities, though today “biqueiras” made from accessible and less porous materials are more common, thereby increasing oil yield. In both cases, interviewees reported that the dough is kneaded for 10 to 30 minutes two to three times per day, and then reformed. This daily working is conducted until the dough, which starts out pinkish in colour and very oily, becomes dark-coloured, dry, and crumbly. This can last for up to a month, depending on the frequency of kneading and the ambient temperature where the dough is stored. Menezes [7] highlights that if the dough is not kneaded it becomes hard, making oil extraction difficult. Interviewees reported that the leftover dry dough and also the discarded shells (see Section 3.2.3) are usually discarded in plastic bags and collected by the urban rubbish collection. This is in contrast to more remote communities which do not have access to such rubbish collections and therefore dispose of all organic waste in the environment [6]. However, three interviewees in the Fazendinha PA explained that they burn the shells to repel mosquitoes, two interviewees reported burning the dry dough for the same reason, and one uses the dry dough to make soap. Other studies have also reported reusing waste materials from the crabwood oil extraction process, burning shells to repel mosquitoes and using the dough to produce soap or as an insect repellent [6, 8].

The processes of forming of the dough observed and reported in the Fazendinha PA have various aspects in common with those reported from other Amazonian communities. For example, in the state of Amazonas, the dough is formed into a ball, though some extractors also then make a groove in the dough to drain the oil [8], whereas on the island of Juba the dough is commonly put into large wooden “biqueiras” and miriti stalks are placed under the dough such that the dough does not come into contact with the wood of the “biqueira,” and rather than forming shapes the dough remains like a cake [6]. The use of “tipiti,” a type of press made of fibres, typical of the Amazon, to collect the oil from the dough has also been reported in Amazonas and on Juba [6, 8]. There is a third possible extraction method in which the dough is boiled in water, with the less dense oil floating to the top and being removed with a gourd [6]. However, this method is only used when it is not possible to use either of the other two methods.

All interviewees reported that they extract crabwood oil in the shade. Although the heat from the sun promotes faster release of oil from the dough [7, 8], some of the interviewees reported that the oil obtained in this way tends to solidify, leading to a lower quality oil, an opinion which has also been reported elsewhere [6, 8]. In the Fazendinha PA, the high human population density may also contribute to the lack of production of crabwood oil in the sun, owing to the aforementioned social rules around exposing the dough to certain community members (Section 3.2.3). Another common practice is to use cotton wicks in the groove of the dough or a fine netting to reduce input of waste materials into the oil through filtration [8, 19], and four of the interviewees reported this practice. The extracted oil is stored in closed containers of various types and sizes. Four interviewees used only plastic water or soda bottles; six used only glass bottles and three used both materials for oil storage. To maintain the quality of the oil it is recommended that small quantities of crabwood oil be stored in closed, dark (amber) glass recipients and large quantities in dark, plastic jerry cans which are new and have not previously been used to store other materials [30]. Nine interviewees reported that there are different types of crabwood oil, eight of whom recognise the types based on colour (green or yellow), with one interviewee relating this colour difference to the extraction method, stating that oil extracted in the sun is yellow and in the shade is green. The ninth interviewee said that the difference between the oil types is in the scent. These differences in fact usually occur as a result of some change in the extraction process, often when inadequate vessels release pigment into the oil, which is why the best practice is to use inert materials such as nonrecycled, preferably transparent, plastics or nonrusting metals [30, 31].

There is much variation in yield of crabwood oil from artisanal and commercial production processes (Table 4), and in the Fazendinha PA interviewees reported a yield of between 1 and 4 litres of oil per 18 litre container of seeds (~11 kg of seeds, as weighed during direct observations). However, measuring yield was generally not considered important by the interviewees, as the oil is for their own, rather than commercial, use. It is therefore possible that the stated estimates of 1–4 litres are not particularly accurate. Indeed, a study using seeds obtained in várzea forest in the state of Amapá, close to the site of the present study, and a (more efficient) chemical extraction process (using hexane as a solvent) found that on average 0.3484 g of oil was obtained per gramme of dry material [32]. Considering this average value for the oil content, the average weight of fresh seeds of 18.8 g, the weight of dry dough of 12.1 g, and the fact that the shell represents on average 23.5% of the dehydrated seed [33], it can be calculated that for a whole fresh seed, the percentage of oil is around 18.8%. This means that for 11 kg of seeds, in a very efficient process, an average of 2 litres of oil would be obtained. It seems likely therefore that the higher yields declared by interviewees in the present study and in a study in the state of Amazonas [8] can at first be disregarded until further investigations can be made. Indeed, there is much variation in the artisanal oil extraction process between individuals, communities, and regions and therefore many factors which may influence yield. For example, some authors report that when seeds are stored for less time they yield more oil [8, 28]. Studies on oil content of cooked and fermented crabwood seeds, and on the differences in composition of oils extracted by press, by solvents and by artisanal methods, are necessary. Beyond this, experimental studies on the artisanal process to measure yield, oil properties, acidity levels, and drying techniques could also contribute to improved understanding and the design of sustainable exploitation of crabwood for oil production.

### 3.3. Process of Knowledge Transfer

The process of artisanal crabwood oil extraction is a knowledge held solely by members of communities within the Amazonian várzea and terra firme forests in which crabwood trees are found. Owing to differences in culture, environment, and history, the specifics of the extraction process vary between individual communities and regions, as discussed in Section 3.2. Also worth noting is that the process is dynamic and interviewees reported that many innovations are tested. If such innovations work, they become incorporated in the process. However, despite all of this variation, the processes of knowledge transfer are seemingly always informal and rely heavily on observation, though that is not to say that help is not offered, and there is always a knowledgeable person on hand to demonstrate the process. The informality of the knowledge transfer is fuelled by the fact that the extraction process is carried out in a family setting, involving at various stages all members of the family and therefore representing a moment of exchange between family members. Indeed, children usually familiarise themselves with the extraction process very early and through their participation gain skills which allow them to contribute effectively to family activities [6]. The seed shelling stage is probably when most interaction and knowledge transfer between family members occurs, as they are gathered together around the cooked seeds.

In the Fazendinha PA, eight interviewees stated that they learned the crabwood oil extraction process mainly with their mother and/or grandmother, another learned with their mother and mother-in-law, another with their father, and three interviewees reported that they learned by themselves. For example, one interviewee considered that they had learned independently, that no one had taught them as such, but rather that they had learned by observing others without active teaching or an obligation to learn. They further elaborated that their siblings had no interest in learning the process; they would help to collect seeds when asked to by their mother but did not show an interest beyond this and therefore did not dominate the techniques. The results presented here represent a different case in comparison with more isolated communities in that a young person who was born in this peri-urban protected area would have opportunities to learn many different skills, whereas in more isolated communities the impulse to learn such processes may more commonly come from the individual themselves as opposed to familial pressure, as a result of closer contact with the environment, restricted access to more diverse learning opportunities, and necessity, for example, resulting from a lack of access to other medicines. Among the interviewees in the Fazendinha PA only one stated that no one else in their family knew how to extract crabwood oil. Only four interviewees however stated that they had been able to pass this knowledge on to the next generation through their children. The others listed their mothers and siblings as other family members who also dominate the techniques involved in the process. Beyond this, two of the interviewees are young (aged between 25 and 30) and have small children, who still have the potential to learn the techniques.

### 3.4. Uses of Crabwood Oil

Following the information provided by the 13 interviewees, in the Fazendinha PA crabwood oil is mainly used as a medication, principally to treat illnesses of the respiratory system and inflammation, though it is also used as an insect repellent and for aesthetic purposes as a hair conditioner (Table 1). Other studies have also shown that medicinal uses are the main uses of crabwood oil, followed by cosmetic uses [6, 8, 48]. Owing to its traditional use as anti-inflammatory, to aid in wound healing and as an insect repellent, the oil has drawn scientific interest in studying its medicinal properties. Furthermore, some researchers have hypothesised that the artisanal oil contains some different active substances as a result of the fermentation process [7, 28, 29] or that the artisanal process leads to greater availability of oil as a result of changes in the structure of the cell walls following the resting period [8]. It is probably the importance of this oil for medicinal use which has perpetuated the artisanal extraction of crabwood oil in this peri-urban setting.

In the majority of published studies on the actions of components of crabwood oil, the oil used is industrialised, usually extracted in a press and refined (Table 2). Just one study reported on the use of artisanal oil, and in another it was not possible to identify the extraction method. There is also scientific interest in the chemical components of the oil, especially in the limonoid-rich fraction, substances present in the Meliaceae which have a bitter taste, which are highly biologically active and to which the main medicinal properties of crabwood oil are attributed [49] (Tables 2 and 3). There are many substances in the limonoid fraction of crabwood oil with possible biological actions. Some have been tested and there are probably others which have yet to be tested or even identified. There are many possible uses and ever more studies developing techniques to make the use of the oil more efficient. For example, Senhorini et al. [50] produced polymeric micro particles containing crabwood oil, and Ferreira et al. [51] used nonionic surfactants to emulsify the oil.

## 4. Conclusion

In the Fazendinha PA, the process of artisanal crabwood oil extraction consists basically of four stages: seed collection; seed cooking and resting; shelling and dough preparation; and finally collection of the oil. Each stage follows a general pattern and is influenced by social rules surrounding which community members can be involved in each stage of the process; however there are variations in the process between individual extractors and communities. Crabwood oil production is a family process and commercialisation is minimal, occurring only between neighbours.

The process of artisanal oil extraction in this area has been maintained mainly as a result of the medicinal properties of the oil. Indeed, artisanal crabwood oil extraction is so important for riverine communities in the Amazon that it persists even in peri-urban areas. However, the difficulty of accessing crabwood seeds as a result of a lack of clear understanding of the rules around use of natural resources in the protected area puts the continuation of this activity at risk. Appreciation of traditional knowledge, as well as scientific, technological, and market research in the area, and relevant public policy and or state and federal government programs can help to promote the extraction of crabwood oil and other oilseeds from várzea forest and support the continuation of artisanal processes. The information gaps to improve the process should be filled with new studies and an appreciation of sustainable and artisanal products encouraged through diverse mechanisms, such as seals, to help foster commercial production of the oil.

## Figures and Tables

**Table 1:** Absolute and relative frequency of uses of crabwood oil among community members in the Fazendinha PA, Macapá, Amapá, Brazil.

Crabwood oil use	Absolute frequency	Relative frequency
Sore throat treatment	12	92%
To treat bruises	7	54%
Massage to relieve body pain	6	46%
To treat wounds	3	23%
Insect repellent	2	15%
Hair conditioner	2	15%
Cough treatment	2	15%
Abdominal massage in pregnant women	1	8%
Flu treatment	1	8%
Soap-making	1	8%
Asthma treatment	1	8%
To treat swelling	1	8%
“Naval treatment”	1	8%
To make a syrup	1	8%
To treat rheumatism	1	8%

**Table 2:** Studies reporting actions of crabwood oil and its components in various biological processes.

Studies	Origin of the oil	Results
Penido et al. (2005) [34]	Brasmazon, Pará, Brazil	Anti-inflammatory action in the fraction of oil rich in tetranortriterpenoids
Ambrozin et al., (2006) [35]	Commercial oil bought in the city of Belém	Moderate insecticidal action on *Atta sexdens rubropilosa*
Ferraris et al. (2012) [36]	Gedunin (limonoid present in crabwood oil) with 95% purity, from the “Laboratório de produto natural Farmanguinhos”	Antiallergic action of the substance gedunin, present in crabwood oil
de Souza Chagas et al. (2012) [37]	WNF Industry and Comerce Ltd.	Not effective in combating the tick *Rhipicephalus* (Boophilus) *microplus* at the dilutions used in the study.
Farias et al. (2012) [38]	Beraca Sabará Químicos e Ingredientes, São Paulo/Brazil, Lot 05083140ST	Potential in control of ticks *R.* (*B*.) *microplus*, *A. nitens,* and *R. sanguineus*, by interfering with reproduction.
Vendramini et al. (2012) [39]	Farmácia de Manipulação, Drogaria e Homeopatia Art.-Fármacos, Rio Claro, São Paulo, Brazil.	Potential acaricide for *Rhipicephalus sanguineus*
Prophiro et al. (2012) [40]	Seed extract	Showed insecticide action for *Aedes aegypti*
de Barros et al. (2012) [41]	Beraca, Sabará, São Paulo, Brazil.	Potential in control of cat louse (*Felicola subrostratus*)
Miranda Júnior et al. (2012) [42]	Artisanal oil obtained in the lab, using doughs from the community of Alto Tocantins.	Antiplasmodial activity, of both the oil and the fraction rich in limonoids.

**Table 3:** Studies presenting limonoids that are components of crabwood oil. “X” indicates the presence of that chemical component in the results of the study and “—” indicates the absence.

Publications	Origin of the oil	Methyl angolensate	7-Deacetoxy-7-oxogedunin	7-Deacetylgedunin	6*a*-Acetoxygedunin	Gedunin	Andirobin	1,2-Dihydro-3b-hydroxy-7-deacetoxy-7-oxogedunin	6-Hydroxy-methyl angolensate	17-Hydroxyazadiradione	Xyloccensin k	6*α*-Acetoxy-7-deacetilgedunin (pericarp)	Methyl angolensate (pericarp)
Ambrozin et al. (2006) [35]	Commercial oil bought in Belém city, state of Pará, Brazil	X	X	—	X	X	—	X	—	X	X	—	—
da Silva et al. (2009) [43]	Extracted by solvent	X	X	X	X	X	X	—	—	—	—	—	—
Miranda Júnior et al. (2012) [42]	Artisanal oil obtained in the lab from doughs produced in Alto Tocantins	—	X	X	X	X	X	X	—	—	—	—	—
Silva et al. (2012) [44]	Extracted by solvent and isolated from the pericarp	—	X	X	X	—	—	—	X	—	—	X	X

**Table 4:** Crabwood oil yield calculated according to our results and data found in the literature. The yield was transformed into kg, using an oil density of 0.925 [33].

Studies	Location/forest type	Mode of oil extraction/method of obtaining information	Seeds (kg)	Oil (kg)	Yield% (kg of oil/kg of seeds)
*Present study*	*Fazendinha PA/várzea*	*Artisanal/interview*	*11–2.8*	*0.925*	*8.5–34*
Homma (2003) [45]; Menezes (2005) [7]	Tomé-Açu-PA/terra firme (plantation)	Artisanal/observation (large-scale production)	20–30	0.925	5–3
Mendonça and Ferraz (2007) [8]	Amazonas/várzea and terra firme	Artisanal/interviews	11–2	0.925	8.5–465
Silva et al. (2010) [19]	Flona do Tapajós, PA	Artisanal/interviews	10	0.925	9
Shanley and Medina (2005) [28]	Santarém, PA	Artisanal	40	0.925	2
	Santarém, PA	Artisanal	40	2.775	7
	Cametá, PA	Artisanal	40	5.55	14
Plowden (2004) [46]	Rio Gurupi/terra firme and várzea	Artisanal/interview	14.43	0.925	6.5
Oliveira (2011) [47]	Tucuruí, PA/terra firme	Artisanal/interview	3270	439.8	13.5
Gomes (2010) [33]	South of Amapá/várzea	Press (9 tonne)/experiment	1000	203.2	20
Gomes (2010) [33]	Terra firme	Press (9 tonne)/experiment	1000	158.6	16
Guedes et al. (2008) [26]	Mazagão, AP, várzea	Press (9 tonne)/experiment	—	—	23
